# Identification of Sarin Simulant DMMP Based on a Laminated MOS Sensor Using Article Swarm Optimization-Backpropagation Neural Network

**DOI:** 10.3390/s25092734

**Published:** 2025-04-25

**Authors:** Ting Liang, Yelin Qi, Shuya Cao, Rui Yan, Jin Gu, Yadong Liu

**Affiliations:** 1 Institute of NBC Defence, Beijing 102205, China; 18511090259@163.com (T.L.); fhqiyelin@163.com (Y.Q.); yrqd77@sohu.com (R.Y.); gj8410@126.com (J.G.); 2 State Key Laboratory of Chemistry for NBC Hazards Protection, Beijing 102205, China; caoshuya123456@126.com

**Keywords:** MOS sensor, DMMP, PSO-BPNN, selectivity, identification

## Abstract

A Pt@CeLaCoNiOx/Co@SnO_2_ laminated MOS sensor was prepared using Co@SnO_2_ as the gas-sensitive film material and Pt@CeLaCoNiOx as the catalytic film material. The sensor was verified to exhibit good sensing performances for dimethyl methylphosphonate, a simulant of Sarin, under a temperature modulation, and characteristic peaks appeared in the resistance response curves only for dimethyl methylphosphonate. The Article Swarm Optimization-Backpropagation Neural Network had a good ability to identify the resistance response data of dimethyl methylphosphonate. The identification accuracy increased as the concentration of dimethyl methylphosphonate increased. This scheme can effectively identify whether the test gas contained dimethyl methylphosphonate or not, which provided a reference for achieving the high selectivity of the MOS sensor for Sarin.

## 1. Introduction

Sarin (GB), as a nerve agent, is a common chemical weapon of mass destruction in the battlefield environment [1]. Common detection techniques for GB include gas chromatography, Raman spectroscopy, ion mobility spectrometry, etc. [2]. However, these devices are usually relatively large and complex to operate, and sometimes, it is difficult to fulfill the testing requirements of GB.

MOS sensors have the advantages of fast response, low power consumption, low price, long life, easy miniaturization and portable use, which have become a popular research direction for gas sensors in recent years [3,4,5,6,7,8]. MOS sensors for the detection of GB are of great research value, and some progress has been made. Due to the specificity of GB, experiments were mainly carried out with Dimethyl methylphosphonate (DMMP) instead of GB, both of which have similar chemical structures. Yoo [9] doped Al into ZnO NPs, and the modified Al-ZnO material showed a fast response time and high resistance response to DMMP. Alali [10] prepared a gas-sensitive material with a heterogeneous structure of WO_3_/CuO. The material had sufficient oxygen vacancies to absorb and desorb oxygen ions and showed a good resistance response to DMMP at 0.5 ppm at room temperature.

Yet poor selectivity has long been a major constraint on the development of MOS sensors. Various methods have been tried to improve the selectivity of MOS sensors for target gases, including the construction of multidimensional nanostructures, precious metal decoration and doping, temperature modulation, heterostructures, carbon nanomaterials, heating, UV irradiation, etc. [11,12,13]. In recent years, a lot of attention has been paid to the construction of sensing materials with laminated structures based on the original gas-sensitive materials covered with additional functional materials. Alali [14] prepared laminated sensing materials for the detection of DMMP by covering the surface of Reduced Graphene Oxide (rGO) with Co_3_O_4_. Due to the hydrogen bonding between DMMP molecules and Co_3_O_4_, the rGO/Co_3_O_4_ showed good selectivity to DMMP. However, the currently proposed schemes were mainly based on the high resistance response of MOS sensors to DMMP to evaluate their high selectivity, which is easily affected by factors such as complex gases, duration of use, humidity, etc., so a really effective solution has not been found yet.

In order to further enhance the identification capability and intelligence of MOS sensors for target gases, combining classification models and sensors has proved to be an effective approach [15]. Currently, models such as Artificial Neural Networks (ANN), Support Vector Machines (SVM) and Nearest Neighbours (KNN) have been validated to be used for the identification of target gases by sensors, and the models optimized by the algorithms can further improve the accuracy of the identification [16].

In this paper, the characteristic peaks of DMMP were obtained based on Pt@CeLaCoNiOx/Co@SnO_2_ sensors, and Article Swarm Optimization-Backpropagation Neural Network (PSO-BPNN) was utilized to categorize and identify DMMP under gas interference, which verified that the neural network had a good capability of identifying the characteristic peaks of resistance response. This achievement can provide certain references for the pattern identification of single-channel MOS sensors and provide technical support for the next step to improve Sarin’s selectivity and intelligence.

## 2. Materials and Methods

### 2.1. Preparation of Film Materials

#### 2.1.1. Preparation of Co@SnO_2_ Slurry

The manufacturing method of the Co@SnO_2_ material was as follows: 6 mmol SnCl_4_·5H_2_O was dispersed in 50 mL benzyl alcohol and stirred for 1 h to obtain the precursor, which was reacted at 160 °C for 24 h. After drying, it was washed twice with a mixture of anhydrous ethanol and dichloromethane and twice with pure water, then put into an oven to be burned at 450 °C for 2 h to obtain the SnO_2_ matrix powder. Dispersed 2 g SnO_2_ powder in 20 mL anhydrous ethanol, added 0.5 wt% 0.01 g/mL Co(NO_3_)_3_·6H_2_O ionic solution in accordance with Co: SnO_2_, stirring for 30 min, and put it into the oven to dry at 60 °C. The modified SnO_2_ powder was obtained using the above steps. 3 g Co@SnO_2_ powder was added to 9 mL nano-alumina dispersion, and ball milled at 400 rpm for 4 h to obtain Co@SnO_2_ slurry. All of the above reagents are from Aladdin Chemical Reagent Co., Ltd., Shanghai, China.

#### 2.1.2. Preparation of Pt@CeLaCoNiOx Slurry

The manufacturing method of the Pt@CeLaCoNiOx material was as follows: 1 g γ-Al_2_O_3_ and 0.5 wt% 0.01 g/mL H_2_PtCl_6_·6H_2_O ionic solution, 10 mol% CeO_2_, La_2_O_3_, Co_3_O_4_, NiO powders (powders were purchased from Nanjing Xian Feng Nanomaterials Technology) were added to the ball milling jars in accordance with the Pt (Ce, La, Co, Ni): Al_2_O_3_, and then 10 mL anhydrous ethanol was added to the jar. The ball milling was performed at 400 rpm for 4 h. The Pt@CeLaCoNiOx slurry was obtained using the above steps.

### 2.2. Preparation of the Catalytic Film/Gas Sensitive Film MOS Sensor

**Figure 1:**
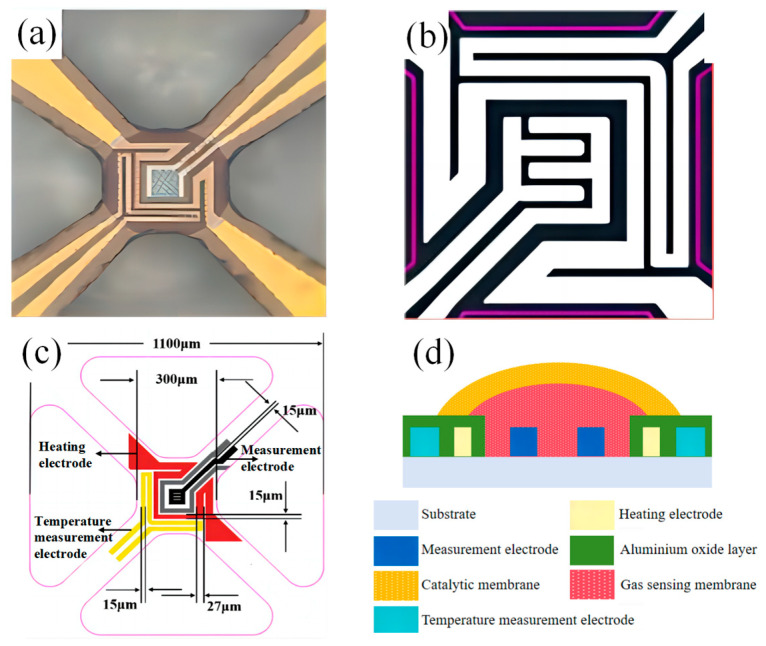
(**a**–**c**) Schematic diagrams and (**d**) physical diagram of the sensor chip.

Figure 1a–c shows the arrayed MOS sensor chip developed and designed by our team, where Figure 1a shows the physical diagram of the chip and Figure 1b shows the electrode design of the chip. The electrodes are specifically divided into heating electrodes, temperature measurement electrodes and measurement electrodes, whose distribution positions and size parameters are shown in Figure 1c. The substrate is zirconium oxide with good mechanical strength. Since the MOS material is affected by the working temperature, we have chosen Pt as the heating electrode with good thermal stability and good linearity between temperature and resistance. In order to obtain the resistance change of MOS material more quickly and accurately, we chose Au with lower resistivity as the measurement electrode.

Using the electrofluidic microspray process, a 60 µm aperture needle is first sprayed with Co@SnO_2_ slurry at the electrode site on the zirconia microthermal plate chip. In order to prevent the interpenetration between the gas-sensitive film and the catalytic film, the gas-sensitive film is dried at 80 °C after spraying. Then, the Pt@CeLaCoNiOx slurry is sprayed using a 100 μm needle to ensure that the gas-sensitive film is completely covered, as shown in Figure 1d. Finally, the chips, after spraying the catalytic film slurry, are put into a muffle furnace at 350 °C and 550 °C for 2 h each to obtain the Pt@CeLaCoNiOx/Co@SnO_2_ laminated sensor.

### 2.3. Test Platform

**Figure 2:**
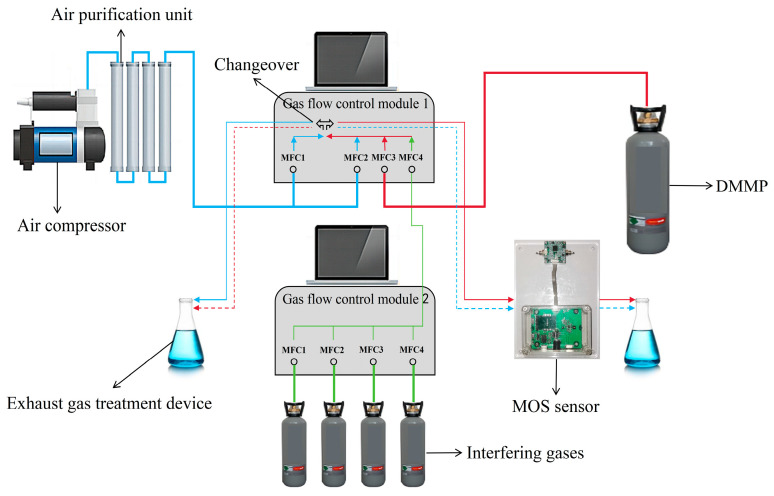
Static generating device.

The test platform is shown in Figure 2. Air is supplied by an air compressor, and through the air purification device, the purified air is divided into two paths, MFC1 and MFC2 (referred to as 1-MFC1 and 1-MFC2), in the gas flow control module 1. DMMP is supplied by gas cylinders and enters MFC3 (referred to as 1-MFC3) in the gas flow control module 1. The interfering gases are supplied by gas cylinders, and the interfering gases enter MFC1, MFC2, MFC3, and MFC4 (referred to as 2-MFC1, 2-MFC2, 2-MFC3, and 2-MFC4) in the gas flow control module 2. Each of the above gases can be adjusted for flow size. The interfering gases are mixed after passing through 2-MFC1, 2-MFC2, 2-MFC3, and 2-MFC4, and the mixed interfering gases enter MFC4 (referred to as 1-MFC4) in the gas flow control module 1. After mixing, 1-MFC2+1-MFC3+1-MFC4 will be formed into the testing path, and 1-MFC1 will be the air path. Switching of the testing path and air path can be realized by controlling the changeover.

Considering that humidity has a large effect on MOS sensors, it is not conducive to accurate performance testing. Therefore, sufficient desiccant is added to the air purification unit in the test platform. In addition, the test units of the MOS sensors are all in closed pipelines to avoid the influence of water and other interfering gases in the air on the performance of the MOS sensors.

## 3. Results and Discussion

### 3.1. Characterization of Material

**Figure 3:**
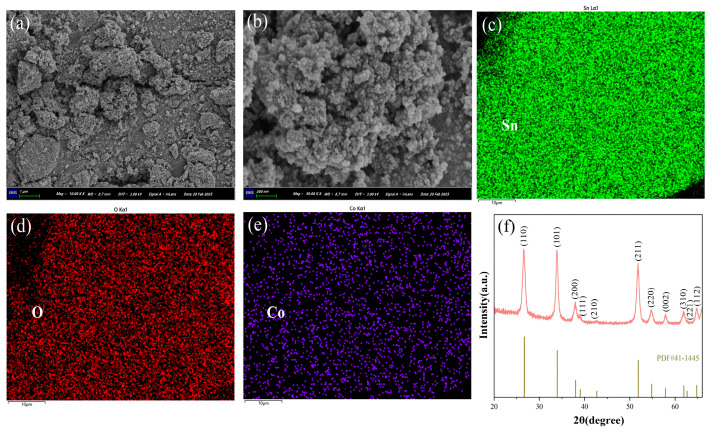
(**a**,**b**) SEM images, (**c**–**e**) EDS images and (**f**) XRD pattern of Co@SnO_2_ powder.

Figure 3a,b shows the SEM images of Co@SnO_2_ powder. As can be seen, Co@SnO_2_ is formed by the accumulation of a large number of uniform nanoparticles; the diameter of the synthesized Co@SnO_2_ nanoparticles is about 20 nm. Figure 3c–e shows the EDS images of Co@SnO_2_ powder; the major elements Sn, O and Co were evenly distributed within the powder.

Figure 3f shows the XRD pattern of Co@SnO_2_ powders. It shows that the Co@SnO_2_ has good purity and crystallinity, with almost no additional impurity peaks. SnO_2_ can be indexed to the corresponding standard card (41-1445), but due to the small amount of Co^3+^ modification, it is difficult to see the relevant information of modified elements from the figure.

### 3.2. Sensing Performance

**Figure 4:**
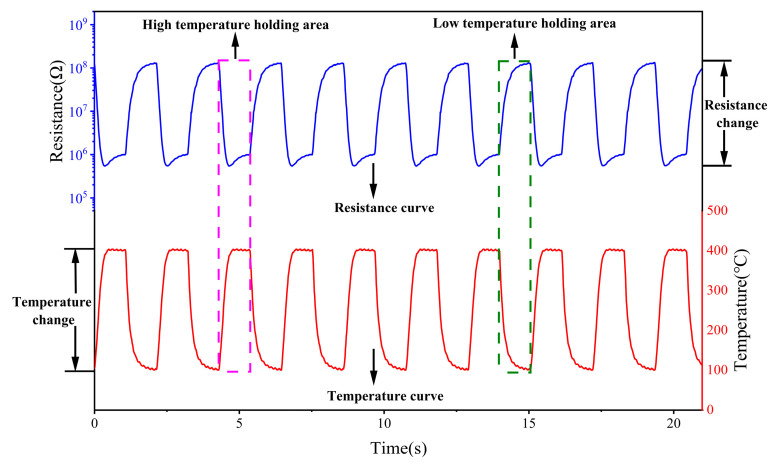
The detection principle of periodic temperature modulation for the sensor.

In this paper, we use a rectangular wave-based temperature dynamic modulation mode, which has been shown to help improve the sensor’s selectivity for the target gas [17], as shown in Figure 4. Compared to the temperature dynamic modulation mode of triangular and sinusoidal waves, this modulation mode has a high-temperature retention phase and low-temperature retention phase, where the MOS material can desorb the gases remaining on the surface of the material during the high-temperature retention phase and adsorb reactive oxygen species during the low-temperature retention phase thereby enhancing the response to the toxic agent. Such a heating mode is suitable for the detection of chemical warfare agents.

**Figure 5:**
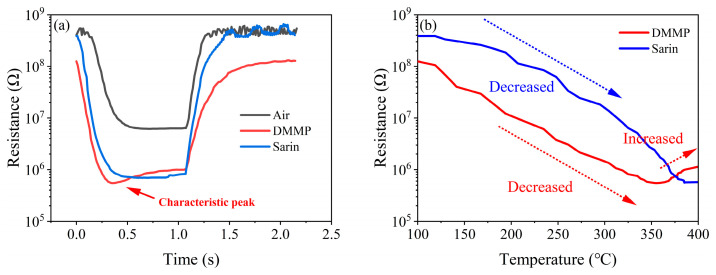
(**a**) Resistance response of air and DMMP in a single cycle, (**b**) Functional relationship between temperature and resistance in DMMP resistance response.

Figure 5a demonstrates the resistance response of the Co-SnO_2_/CeLaCoNiOx sensor to air and 1 mg/m^3^ DMMP under the above modulation mode, where the high temperature was 400 °C, low temperature was 100 °C, high temperature holding time and low temperature holding time was 1 s. A characteristic peak can be observed in the resistance response of DMMP compared to air. A characteristic peak can be observed in the resistance response of DMMP compared to air and Sarin.

Figure 5b demonstrates the function of temperature and resistance, where the resistance response of DMMP showed a decreasing trend at 100–350 °C and an increasing trend at 350–400 °C, leading to the characteristic peak. Sarin’s resistance response consistently showed a decreasing trend in the heating area.

The first point to be clarified is that the catalytic film material has good catalytic activity for DMMP. Under the combined catalytic effect of CeO_2_ [18], La_2_O_3_ [19], Co_3_O_4_ [20] and NiO [21], DMMP would undergo rapid demethylation to produce phosphorus-containing intermediates and localized methyl oxidation to produce small-molecule aldehydes and alcohols. During the preparation of the catalytic film material, after H_2_PtCl_6_, 6H_2_O was dissolved as the precursor, the Pt^4+^ ions would be bonded to the active sites on the surface of γ-Al_2_O_3_ or inside by physical adsorption or chemical action. During the ball milling process, some Pt^4+^ ions would be reduced to Pt atoms into the γ-Al_2_O_3_ lattice, which increased the specific surface area and active sites of γ-Al_2_O_3_ and enhanced the catalytic activity of the material for DMMP [22].

When the temperature of the catalytic film was lower than 350 °C, the catalytic efficiency of the catalytic film for DMMP was very low, and the concentration of DMMP through the catalytic film gradually increased, resulting in a gradual decrease in resistance. When the temperature of the catalytic film was higher than 350 °C, the catalytic film began to have high catalytic efficiency for DMMP; the concentration of DMMP after passing through the catalytic film decreased rapidly, and the concentration of catalytic products increased rapidly. Since the resistance response of the gas-sensitive film to DMMP is much higher than that of its catalytic product, it resulted in a rapid recovery of the resistance, i.e., the appearance of the characteristic peak.

Although the gas-sensitive film also showed a good resistance response to Sarin, the characteristic peaks did not appear. We speculate that this is caused by the poor catalytic efficiency of the catalytic film for Sarin or the proximity of the resistance response of the gas-sensitive film to Sarin and its catalytic product.

**Figure 6:**
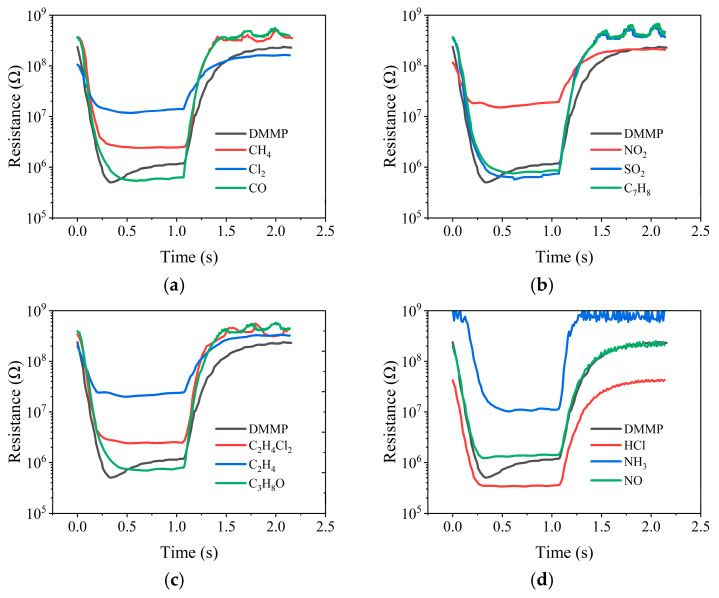
(**a**–**d**) Resistance responses to DMMP and 12 interfering gases.

We took the 12 interfering gases as the control and performed the same test under the above-mentioned operating conditions. The results are shown in Figure 6. From Figure 6a–d, it can be seen that the sensor only produced a characteristic peak in the resistance response curve to DMMP, indicating that it had good selectivity to DMMP.

**Figure 7:**
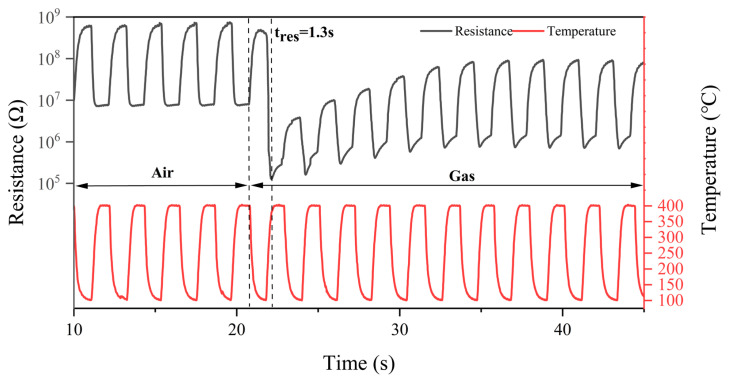
Response time of the sensor for DMMP.

Response time is one of the properties to be focused on for chemical warfare agent sensors. In the temperature dynamic modulation mode described above, we first placed the sensor in the air, waited until the resistance stabilized, and switched to 0.5 mg/m^3^ DMMP, as shown in Figure 7. From the figure, it can be found that the resistance of the sensor can respond quickly to the change in temperature, and the response time can be up to 1.3 s.

**Figure 8:**
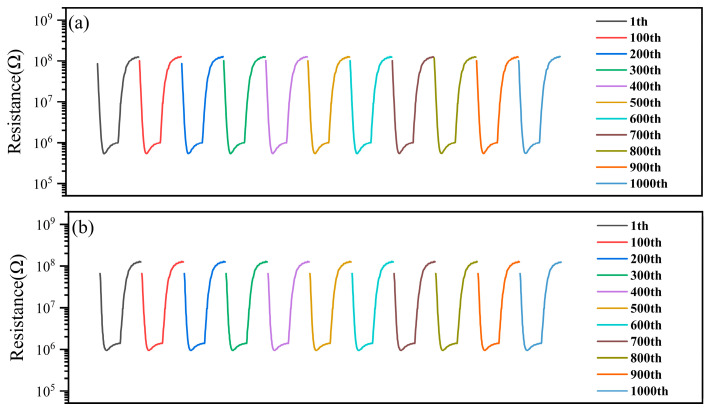
(**a**) Stability of the sensor for DMMP and (**b**) 130 days later.

In order to test the stability of this sensor under the above temperature dynamic modulation, we continuously tested the sensor 1000 times to 0.5 mg/m^3^ DMMP and recorded the resistance response curves (1 pulse recorded every 100 times). The test results are shown in Figure 8a. It can be seen that the resistance curve of the 1000th time was not significantly different from that of the first time, and there was almost no change to the characteristic peak of DMMP. The same test was performed again after 130 days, and it can be seen that the characteristic peaks of the sensor for DMMP have slightly decreased, but still, a clear characteristic peak can be observed, as shown in Figure 8b, which indicated that the sensor was of good stability.

## 4. Definition of Peak Height

### 4.1. Sample Data Collection and Categorisation

**Figure 9:**
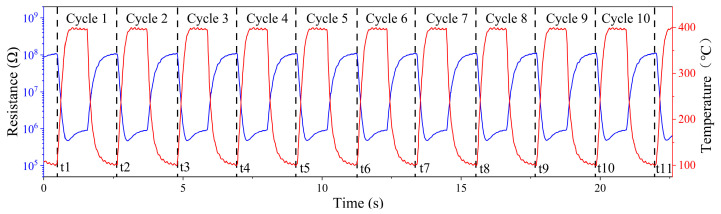
Process of Sample Data Collection.

Based on the above temperature modulation, the temperature change and resistance change were periodic. At the end of the low-temperature hold, the test system automatically recorded the time (ti) at that time. As shown in Figure 9, when the first low-temperature hold ended after the test had started, the test system recorded the time point at that time as t1 and the second time as point t2 and kept recording like this until the end of the test. Therefore, the resistance data between two adjacent time points can be considered as a cycle in the periodic resistance data. In this temperature modulation, the test system will record 150 resistance values fixedly in each cycle, and the 150 resistance values in each cycle can be treated as one sample data. Each of the 150 resistance values in a sample was used as a feature of the sample, so the number of features in the neural network is 150.

The samples detected by the sensor were categorized into a total of four categories, namely air, air+DMMP, air+interfering gas, and air+DMMP, interfering gas, which were numbered 1, 2, 3, and 4, respectively. The concentration of DMMP and 12 interfering gases are all 0.5 mg/m^3^. A total 200 samples were collected for each category of gas, forming a dataset (800 × 150), as shown in Table 1. Based on pattern recognition, different gas categories will be identified. Such a gas categorization allows for simultaneous verification of the scheme’s ability to identify DMMP and anti-interference [23].

### 4.2. Prediction of Gas Categories Based on the PSO-BP

**Figure 10:**
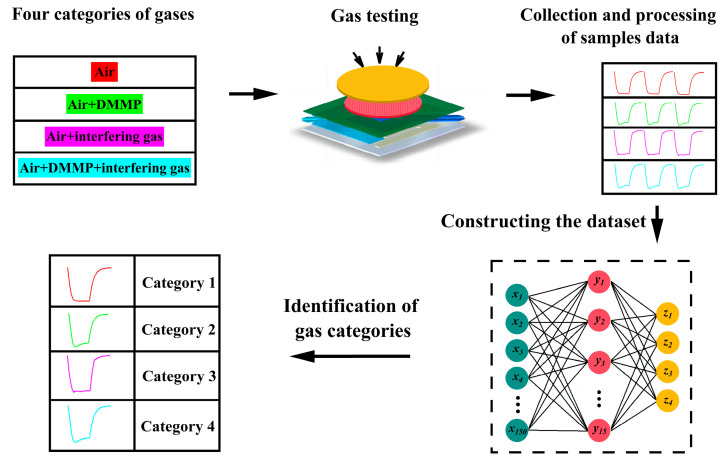
The sensor is combined with the neural network.

Figure 10 shows the process of combining the Co@SnO_2_/Pt@CeLaCoNiOx sensor with a neural network to predict gas categories. There are three main processes: sample data collection, data processing and identification of gas categories. Firstly, the sensors were used to collect the resistance response to the four categories of gases, and then the features were extracted to construct the dataset. Finally, the neural network was used to predict the gas categories.

BPNN (Back propagation neural network) [24] is a multifilm feed-forward neural network trained according to the error back propagation algorithm [25,26,27]. Through the implementation of function approximation, pattern identification, classification, data compression and other functions, it is widely used in the field of image, speech and modulation identification, etc [28]. However, because the BPNN only changes the connection value and threshold of the network and does not change the topology of the network, the BPNN also exists in dealing with specific problems such as network paralysis, slower convergence of network learning, and easy-to-fall into the local minima [29].

In order to solve the above problems, heuristic learning methods were proposed based on numerical optimization and other improvement algorithms; these optimization algorithms, to varying degrees, to improve the learning speed of the BPNN, accelerate the convergence of the network, to avoid falling into the local minima [30,31].

**Figure 11:**
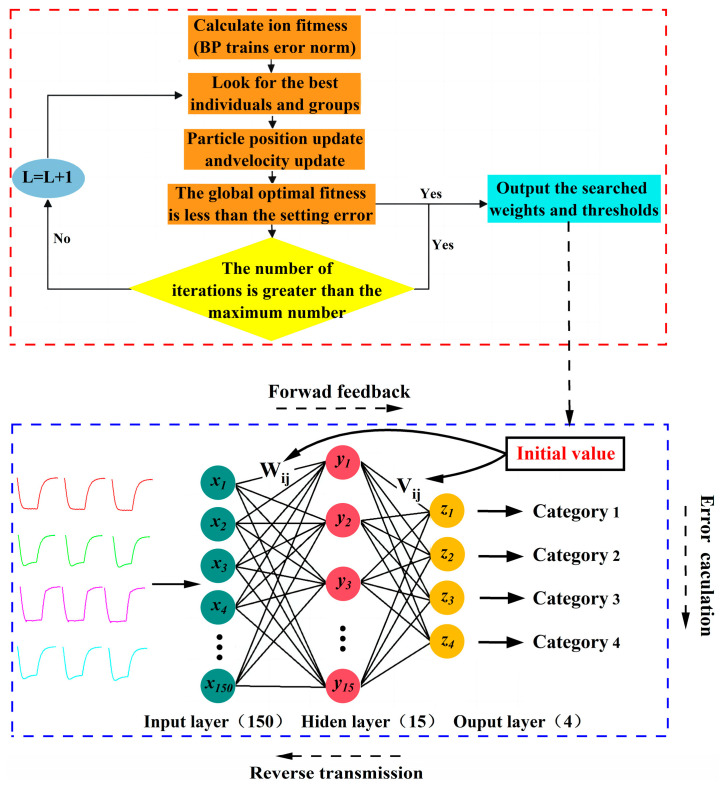
Optimization of BPNN by PSO.

The PSO algorithm is a group intelligence optimization algorithm proposed based on the law of birds group collaboration mechanism [32,33]. The PSO algorithm has a strong global search ability, so it is used to optimize the BPNN to make up for the defects of randomly generated weights and thresholds that are prone to fall into local optimums [34]. Velocity and position are two core elements of the PSO algorithm. The key to the algorithm is that each particle has a velocity to determine the direction and distance that they fly. These particles will follow the current optimal particle to search the solution space and find the optimal solution by iteration [35]. The optimal solution optimized by the PSO algorithm is then input to the BPNN as the initial weights and thresholds of the BPNN [36], as shown in Figure 11.

In the constructed dataset, the order of the samples was randomly sorted, and 75% of the samples were used as the training set and the remaining 25% as the test set. In the experiment, we performed the initialization parameters of the PSO algorithm.

According to the empirical formula, the number of hidden layers is usually between the number of input layers and the number of output layers, and too many hidden layers may lead to the overfitting of the data by the BPNN, so we set the hidden layers nodes in order to 5, 10, 15, 20, 25. Since the mean square error is an important index for evaluating the BPNN, a smaller mean square error means that the BPNN has a better prediction accuracy and fitting degree. Meanwhile, the detection of toxic agents needs to be completed in a very short time, so we compared the mean square errors (MSE) and prediction times of PSO-BPNN with different hidden layers nodes.

**Figure 12:**
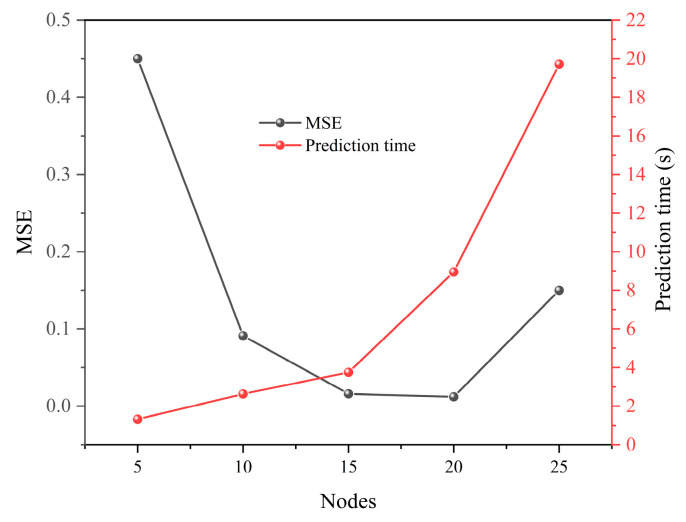
Effect of the hidden layer nodes on the mean square error and prediction time.

As shown in Figure 12, PSO-BPNN simultaneously had a smaller mean square error and shorter prediction time when the hidden layers nodes were 15, so we determined the architecture of PSO-BPNN to be 150-15-4.

**Figure 13:**
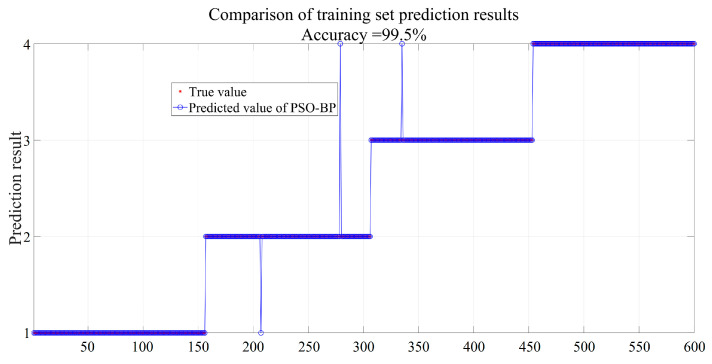
Identification results of PSO-BPNN on the training set.

Figure 13 shows the result of the trained PSO-BPNN. It can be seen that the identification accuracy of the PSO-BPNN on the training set samples reached 99.5%, indicating that the model possessed a high degree of fit to the training data, but its generalization ability still needs to be verified in the identification of the test set.

**Figure 14:**
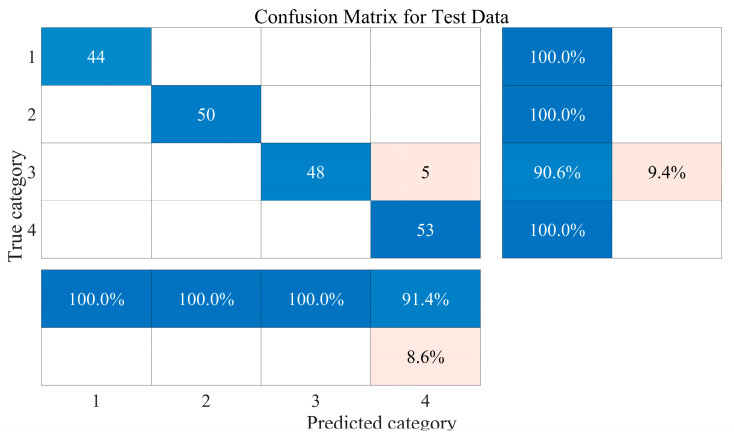
Confusion matrix for test data.

Figure 14 shows the identification results of PSO-BPNN on the test set. Based on the confusion matrix, further analysis of the identification results reveals that PSO-BPNN had 100% accuracy in identifying the gas samples of category 1, category 2, and category 4 and incorrectly identified 5 of the 53 category 3 samples as category 4. This may be due to the high overlap of the resistance response curves corresponding to a few category 3 gas samples with those corresponding to the overall category 4 gas samples.

In order to calculate the accuracy of PSO-BPNN’s identification of DMMP-containing samples, it can be concluded that PSO-BPNN has predicted the number of DMMP-containing samples is 108 (including 50 category 2 samples, 5 category 3 samples, and 58 category 4 samples), and the correct number of them is 103, so the accuracy rate of PSO-BPNN’s identification of DMMP-containing samples is 95.4%, hereafter referred to as PSO-BPNN identification accuracy for DMMP.

### 4.3. Concentration Impact

To further verify the PSO-BPNN identification accuracy for DMMP at different concentrations, we designed the following experiments. With the composition and concentration of the interfering gas unchanged, additional samples of category 2 and category 4 with DMMP concentrations of 0.1 mg/m^3^, 0.3 mg/m^3^, 1 mg/m^3^ and 2 mg/m^3^ were collected. Similarly, four additional datasets were constructed following the construction of the dataset in Table 2. The five datasets are shown in Table 2, and the PSO-BPNN identification accuracy for DMMP was obtained based on the above-mentioned scheme, respectively.

**Figure 15:**
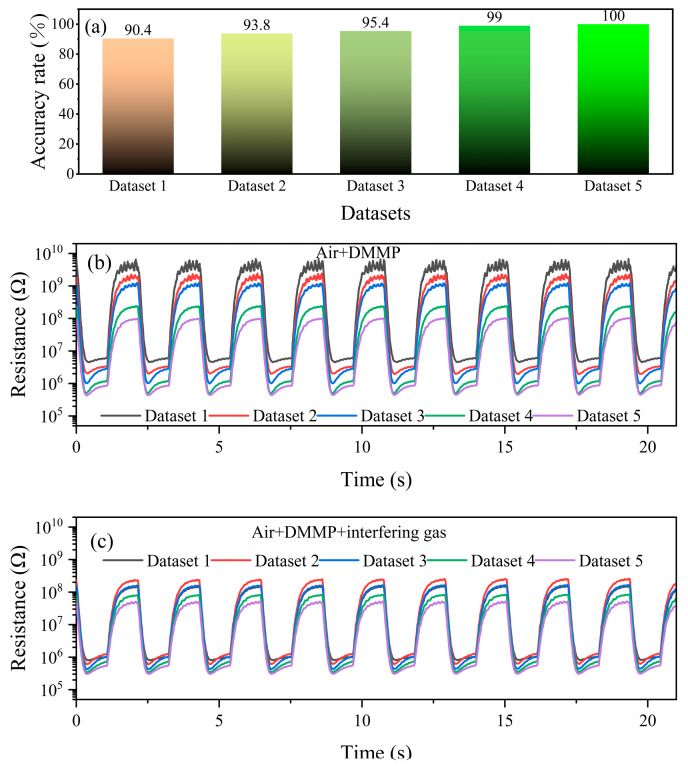
(**a**) The PSO-BPNN identification accuracy for DMMP in different test sets; Resistance response curves of the sensor for (**b**) category 2 and (**c**) category 4 in the 5 datasets.

From Figure 15a, it can be obtained that the PSO-BPNN identification accuracy for DMMP increased with the increase in DMMP concentration. When the DMMP concentration was 2 mg/m^3^, the accuracy was 100%, and even when the DMMP concentration was as low as 0.1 mg/m^3^, the accuracy could still reach 90.4%. It may be due to the fact that as the DMMP concentration increased, the resistance response of the sensor to the DMMP-containing gases became larger, and the characteristic peaks in the response curves also grew, as shown in Figure 15b,c. Such a change in the resistance response leads to an increase in the variability between the characteristics of the gas samples containing and not containing DMMP, which facilitates the identification of the gas samples containing DMMP by the PSO-BPNN.

Based on the above test results, the laminated structure of the Co@SnO_2_/Pt@CeLaCoNiOx laminated MOS sensor combined with the PSO-BPNN can provide effective identification of gases containing DMMP under certain conditions.

## 5. Conclusions

The high sensitivity of the Co@SnO_2_ gas-sensitive film to DMMP and the special catalytic effect of the Pt@CeLaCoNiOx catalytic film on DMMP during the warming process resulted in characteristic peaks in the resistance response of the Pt@CeLaCoNiOx/Co@SnO_2_ sensor to DMMP. Notably, PSO-BPNN is able to accurately recognize the characteristic peaks in the resistance response data, thus enabling accurate identification of DMMP. In our previous work, it has been demonstrated that catalytic film/gas-sensitive film laminated MOS sensors are capable of the presence of characteristic peaks in the resistance response to HCN, a systemically toxic chemical warfare agent [37]. By screening new sensing materials and optimizing the temperature dynamic modulation, the characteristic peaks of the catalytic film/gas-sensitive film laminated MOS sensor to Sarin are very likely to be achieved. Combined with the research results in this paper, it is expected to further enhance the selectivity and intelligence of MOS sensors for Sarin.

## Figures and Tables

**Table 1:** Categorization and amount of samples in the dataset.

Categories	Composition of Gases	Amount of Samples
1	Air	200
2	Air+0.5 mg/m^3^ DMMP	200
3	Air+0.5 mg/m^3^ interfering gas	200
4	Air+0.5 mg/m^3^ DMMP+0.5 mg/m^3^ interfering gas	200

**Table 2:** Categorization and amount of samples in the datasets.

Categories	Composition of Gases	Amount of Samples
Dataset 1		
1	Air	200
2	Air+0.1 mg/m^3^ DMMP	200
3	Air+0.5 mg/m^3^ interfering gas	200
4	Air+0.1 mg/m^3^ DMMP+0.5 mg/m^3^ interfering gas	200
Dataset 2		
1	Air	200
2	Air+0.2 mg/m^3^ DMMP	200
3	Air+0.5 mg/m^3^ interfering gas	200
4	Air+0.2 mg/m^3^ DMMP+0.5 mg/m^3^ interfering gas	200
Dataset 3		
1	Air	200
2	Air+0.5 mg/m^3^ DMMP	200
3	Air+0.5 mg/m^3^ interfering gas	200
4	Air+0.5 mg/m^3^ DMMP+0.5 mg/m^3^ interfering gas	200
Dataset 4		
1	Air	200
2	Air+1 mg/m^3^ DMMP	200
3	Air+0.5 mg/m^3^ interfering gas	200
4	Air+1 mg/m^3^ DMMP+0.5 mg/m^3^ interfering gas	200
Dataset 5		
1	Air	200
2	Air+2 mg/m^3^ DMMP	200
3	Air+0.5 mg/m^3^ interfering gas	200
4	Air+2 mg/m^3^ DMMP+0.5 mg/m^3^ interfering gas	200

## Data Availability

Data are contained within the article.

## References

[1] Emelianova A., Reed A., Basharova E.A., Kolesnikov A.L., Gor G.Y. (2023). Closer Look at Adsorption of Sarin and Simulants on Metal–Organic Frameworks. ACS Appl. Mater. Interfaces.

[2] Witkiewicz Z., Jasek K., Grabka M. (2023). Semiconductor gas sensors for detecting chemical warfare agents and their simulants. Sensors.

[3] Chen F., Yang M., Wang X., Song Y., Guo L., Lu G. (2019). Template-free synthesis of cubic-rhombohedral-In_2_O_3_ flower for ppb level acetone detection. Sens. Actuators B Chem..

[4] Guo L., Chen F., Kou X., Wang C., Sun Y., Lu G. (2018). Ultra-sensitive sensing platform based on Pt-ZnO-In_2_O_3_ nanofibers for detection of acetone. Sens. Actuators B Chem..

[5] Xie J., Wang H., Lin Y., Zhou Y., Wu Y. (2013). Highly sensitive humidity sensor based on quartz crystal microbalance coated with ZnO colloid spheres. Sens. Actuators B Chem..

[6] Bao S., Li K., Ning P., Peng J., Tang L. (2017). Highly effective removal of mercury and lead ions from wastewater by mercaptoamine-functionalised silica-coated magnetic nano-adsorbents: Behaviours and mechanisms. Appl. Surf. Sci..

[7] Wang M., Hou T., Shen Z., Zhao X., Ji H. (2019). MOF-derived Fe_2_O_3_: Phase control and effects of phase composition on gas sensing performance. Sens. Actuators B Chem..

[8] Zhang B., Liu G., Cheng M., Gao Y., Zhao L., Li S., Lu G. (2018). The preparation of reduced graphene oxide-encapsulated α-Fe_2_O_3_ hybrid and its outstanding NO_2_ gas sensing properties at room temperature. Sens. Actuators B Chem..

[9] Patil L.A., Bagul S.D., Patil D.G. (2017). DMMP sensing performance of undoped and al doped nanocrystalline ZnO thin films prepared by ultrasonic atomization and pyrolysis method. J. Nanostruct..

[10] Alali K.T., Liu J., Aljebawi K., Liu P., Chen R., Li R., Wang J. (2019). Electrospun np WO_3_/CuO heterostructure nanofibers as an efficient sarin nerve agent sensing material at room temperature. J. Alloys Compd..

[11] Li Q., Chen D., Miao J., Lin S., Yu Z., Cui D., Chen X. (2021). Highly sensitive sensor based on ordered porous ZnO nanosheets for ethanol detecting application. Sens. Actuators B Chem..

[12] Jiang B., Lu J., Han W., Sun Y., Wang Y., Cheng P., Lu G. (2022). Hierarchical mesoporous zinc oxide microspheres for ethanol gas sensor. Sens. Actuators B Chem..

[13] Yu Z., Gao J., Xu L., Liu T., Liu Y., Wang X., Zhao C. (2020). Fabrication of lettuce-like ZnO gas sensor with enhanced H_2_S gas sensitivity. Crystals.

[14] Alali K.T., Liu J., Chen R., Liu Q., Zhang H., Li J., Wang J. (2019). HFIP-Functionalized Co_3_O_4_ Micro-Nano-Octahedra/rGO as a Double-film Sensing Material for Chemical Warfare Agents. Chem.–A Eur. J..

[15] Wang T., Ma H., Jiang W., Zhang H., Zeng M., Yang J., Yang Z. (2021). Type discrimination and concentration prediction towards ethanol using a machine learning–enhanced gas sensor array with different morphology-tuning characteristics. Phys. Chem. Chem. Phys..

[16] Tang W., Wang J. (2015). Mechanism for toluene detection of flower-like ZnO sensors prepared by hydrothermal approach: Charge transfer. Sens. Actuators B Chem..

[17] Liu Y.D., Zhao S.Y., You L.J., Xu Y., Si R.J., Zhang S.P. (2025). Improving the Selectivity of Metal Oxide Semiconductor Sensors for Mustard Gas Simulant 2-Chloroethyl Ethyl Sulfide by Combining the Laminated Structure and Temperature Dynamic Modulation. Sensors.

[18] Li T., Tsyshevsky R., Algrim L., McEntee M., Durke E.M., Eichhorn B., Karwacki C., Zachariah M.R., Kuklja MMRodriguez E.E. (2021). Understanding dimethyl methylphosphonate adsorption and decomposition on mesoporous CeO_2_. ACS Appl. Mater. Interfaces.

[19] Mitchell M.B., Sheinker V.N., Mintz E.A. (1997). Adsorption and decomposition of dimethyl methylphosphonate on metal oxides. J. Phys. Chem. B.

[20] Sadeghi M., Yekta S. (2016). Adsorption and neutralization chemistry of dimethyl methyl phosphonate (DMMP) as an organo-phosphorous pollutant (OPP) on the surface of nano-structured Co_3_O_4_ and MnCo_2_O_4_ catalysts. Iran. Chem. Commun..

[21] Lee S.C., Choi H.Y., Lee S.J., Lee W.S., Huh J.S., Lee D.D., Kim J.C. (2009). The development of SnO2-based recoverable gas sensors for the detection of DMMP. Sens. Actuators B Chem..

[22] Dhar A., Vekariya R.L., Sharma P. (2017). Kinetics and mechanistic study of n-alkane hydroisomerization reaction on Pt-doped γ-alumina catalyst. Petroleum.

[23] Hosoya Y., Itagaki Y., Aono H., Sadaoka Y. (2005). Ozone detection in air using SmFeO_3_ gas sensor. Sens. Actuators B Chem..

[24] Deng Y., Zhou X., Shen J., Hong H., Lin H., Liao B.Q. (2021). New methods based on back propagation (BP) and radial basis function (RBF) artificial neural networks (ANNs) for predicting the occurrence of haloketones in tap water. Sci. Total Environ..

[25] Zhang J.R., Zhang J., Lok T.M., Lyu M.R. (2007). A hybrid particle swarm optimization–back-propagation algorithm for feedforward neural network training. Appl. Math. Comput..

[26] Masoumi A., Jabari F., Ghassem Zadeh S., Mohammadi-Ivatloo B. (2020). Long-term load forecasting approach using dynamic feed-forward back-propagation artificial neural network. Optimization of Power System Problems: Methods, Algorithms and MATLAB Codes.

[27] Panda S., Panda G. (2020). Fast and improved backpropagation learning of multi-film artificial neural network using adaptive activation function. Expert Syst..

[28] Liu F., Huo W., Han Y., Yang S., Li X. (2020). Study on network security based on PCA and BP neural network under green communication. IEEE Access.

[29] Pan X., Zhou W., Lu Y., Sun N. (2019). Prediction of network traffic of smart cities based on DE-BP neural network. IEEE Access.

[30] Zhang R., Liu M., Yin Y., Zhang Q., Cai Z. (2020). Prediction algorithm for network security situation based on bp neural network optimized by sa-soa. Int. J. Perform. Eng..

[31] Yu F., Xu X. (2014). A short-term load forecasting model of natural gas based on optimized genetic algorithm and improved BP neural network. Appl. Energy.

[32] Fan Z., Zi-xuan X., Ming-hu W. (2023). State of health estimation for Li-ion battery using characteristic voltage intervals and genetic algorithm optimized back propagation neural network. J. Energy Storage.

[33] Huang Y., Zhao R., Cheng Z. (2020). Air quality prediction using improved PSO-BP neural network. IEEE Access.

[34] Li G., Tan Z., Xu W., Xu F., Wang L., Chen J., Wu K. (2021). A particle swarm optimization improved BP neural network intelligent model for electrocardiogram classification. BMC Med. Inform. Decis. Mak..

[35] Abdolrasol M.G., Hussain S.S., Ustun T.S., Sarker M.R., Hannan M.A., Mohamed R., Milad A. (2021). Artificial neural networks based optimization techniques: A review. Electronics.

[36] Mulumba D.M., Liu J., Hao J., Zheng Y., Liu H. (2023). Application of an optimized PSO-BP neural network to the assessment and prediction of underground coal mine safety risk factors. Appl. Sci..

[37] Liu Y., Qi Y., Yang W., Ma T., Zhang S., Liang T. (2025). Improved Selectivity of CeMnOx/Pt@SnO_2_ Laminated MOS Sensor for Hydrogen Cyanide Under Temperature Dynamic Modulation. Nanomaterials.

